# Supplementary material for: Factors affecting clothing purchase intention in mobile short video app: Mediation of perceived value and immersion experience
Source: PLoS One. 2022 Sep 13;17(9):e0273968. doi: 10.1371/journal.pone.0273968 (PMC9469968; doi:10.1371/journal.pone.0273968)
Supplement: Attachment — Submitted filename: 20220408Aquaculture-Lists of Responses.docx [file pone.0273968.s002.docx]

Dear Editors and Reviewers,

Thank you for your patience and kind suggestions on our manuscript. We have revised the Manuscript PONE-D-21-31016 exactly according to the comments, and found these comments are very helpful.

According to the suggestions, we have revised the manuscript according to the comments of the reviewers. We hope this revision can make our paper more acceptable. Special thanks to you for your careful comments. The revisions were addressed point by point below.

**Lists of Responses**

Reviewer #1: 1. Abstract must contain: (a) Originality of the research; (b) Research objective; (c) Method; (d)
Empirical result; (e) Practical implications
Responses: Thanks for your careful revision and thanks a lot for your suggestions. The abstract has been reorganized and I hope this revision will contribute to the article.

2. Provide background information and set the context about your study in the context of research area (country), Introduce the specific topic of your research and explain why it is important, Mention past attempts to solve the research problem or to answer the research question and Conclude the Introduction by mentioning the specific objectives of your research.
Responses: Thanks for your careful revision and thanks a lot for your suggestions. We have rewritten the introduction of the paper, and it has been revised in the text.

3. 3. Specify methods and procedures of your study very clearly, as part of this section you can include research design, variables covered, data analysis method etc.
Responses: Thanks for your careful revision and thanks a lot for your suggestions. We have rewritten the methods and procedures of the paper, and it has been revised in the text.

4. Prove the results and find a linkage among results, research questions and hypothesis by using previous studies/literature review, whether your hypothesis accepted or rejected, matches with similar results and dissimilar. If dissimilar, mention what cause.
Responses: Thanks for your careful revision and thanks a lot for your suggestions. We have linked relevant studies, explain whether they are consistent or inconsistent with our research conclusions, and explain the relevant reasons, and it has been revised in the text.

5. References-Please follow the appropriate referencing system/ of journal’s guidelines accordingly.
Responses: Thanks for your careful revision and thanks a lot for your suggestions. We have revised and reorganized the references of the paper, and it has been revised in the text.

Reviewer #2: This paper explores the relationship between interactivity of social e-commence, perceived value, immersive experience and continuous purchase intention based on the S-O-R framework for fashion products. The authors claim that social e-commence interactivity has a positive effect on consumers’ perceived value, immersive experience and continuous purchase intention of fashion products, perceived value influences immersive experience and continuous purchase intention, and immersive experience positively influences continuous purchase intention. The study is clear and significant. However, I have some concerns about this paper:

1. I doubt the theoretical contribution in the current manuscript. Past researches have shown the relationship between social media interactivity, perceived value, immersive experience and purchase intention (e.g., social media and purchase intention, Chang Ya Ping and Dong 2016; perceived interactivity and value, Li et al. 2021; perceived value and continuance intention, Changlin et al. 2020). So, the present research’s contribution hangs almost wholly on the novelty of the variable, continuous purchase intention. Authors should elaborate more about the concept of continuous purchase intention, especially about its difference from purchase intention.

Responses: Thanks for your careful revision and thanks a lot for your suggestions. In the process of writing this paper, we did not highlight the contribution of this paper, which made this paper too dependent on previous research. In fact, we not only reorganize the variables, but also pay attention to the latest form of social e-commerce. This paper focuses on the clothing purchase intention of mobile short video application. Therefore, this paper has good theoretical value and practical significance for fashion marketing and mobile short video application marketing. Through the guidance of the reviewers, we can highlight the contribution of the paper and better translate the names of variables
2. The managerial application of the current research is limited. We have known that the interactivity of social media has many positive effects, including immersive experience, perceived value and purchase intention. Companies want to sell more products and the increased purchase intention of consumers are beneficial. Therefore, managers can utilize social media and maintain its interactivity. But what is the difference between continuous purchase intention and purchase intention? I advise that authors pay more attention to the suggestions about how to increase the interactivity of social media and the significance of the continuity of purchase intention.

Responses: Thanks for your careful revision and thanks a lot for your suggestions. We highlight the link between media interactivity and continued purchase intention, and it has been revised in the text.
3. I do not think the elaboration of the theory development is specific enough. For H1, social media interactivity has a significant impact on continuous purchase intention, the theory supporting this hypothesis only relies on the research of Chang Ya Ping and Dong (2016), and Gasawneh et al. (2020) who showed the social media positively affect the purchase intention. How the interactivity or social media influences the continuous purchase intention is missing. Authors should explain in detail how the effect of social media is working, i.e., the underlying process. The same as other hypotheses, authors should explain the theory development in more detail.

Responses: Thanks for your careful revision and thanks a lot for your suggestions. We feel that the hypotheses of the manuscript are not very clear, which brings a lot of trouble to the reviewer's review. After the guidance of the reviewer, we are impressed, so we rewrite all the hypotheses, and it has been revised in the text.
4. The research focuses on fashion products. I am wondering how you defined fashion products, and whether it is different from other kinds of products. Since the consumers of the fashion industry tend to have more social needs, interactivity seems to have a greater impact on consumers. Maybe the authors can discuss this in the future research part.

Responses: Thanks for your careful revision and thanks a lot for your suggestions. We apologize for our lax statement. Because our research belongs to the field of fashion marketing, we directly use fashion. In fact, it should be more accurate to use clothing, and it has been revised in the text.
5. The whole writing should be polished, as there are several grammar errors in the manuscript.
Responses: Thanks for your careful revision and thanks a lot for your suggestions. We have invited native English editors to revise the grammar and expression of this paper. I hope this revision can make the expression of this paper more accurate, and it has been revised in the text.
