# Supplementary material for: Factors affecting clothing purchase intention in mobile short video app: Mediation of perceived value and immersion experience
Source: PLoS One. 2022 Sep 13;17(9):e0273968. doi: 10.1371/journal.pone.0273968 (PMC9469968; doi:10.1371/journal.pone.0273968)
Supplement: Attachment — Submitted filename: 20220616Response to Reviewers.docx [file pone.0273968.s003.docx]

Dear Editors and Reviewers,

Thank you for your patience and kind suggestions on our manuscript. We have revised the Manuscript PONE-D-21-31016 exactly according to the comments, and found these comments are very helpful.

According to the suggestions, we have revised the manuscript according to the comments of the reviewers. We hope this revision can make our paper more acceptable. Special thanks to you for your careful comments. The revisions were addressed point by point below.

**Lists of Responses**

1. The managerial implications of this paper could be further elaborated.

Responses: Thanks for your careful revision and thanks a lot for your suggestions. We have further elaborated the managerial implications of this paper, and it has been revised in the text.
2. A figure with the mediation analysis might be provided.

Responses: Thanks for your careful revision and thanks a lot for your suggestions. We have made a figure with the mediation analysis (figure 2), and it has been revised in the text.
